# Gender differences in the association of visceral and subcutaneous adiposity with adiponectin in African Americans: the Jackson Heart Study

**DOI:** 10.1186/1471-2261-13-9

**Published:** 2013-02-22

**Authors:** Aurelian Bidulescu, Jiankang Liu, DeMarc A Hickson, Kristen G Hairston, Ervin R Fox, Donna K Arnett, Anne E Sumner, Herman A Taylor, Gary H Gibbons

**Affiliations:** 1 Department of Community Health and Preventive Medicine, Cardiovascular Research Institute, Morehouse School of Medicine, 30310, Atlanta, GA, USA; 2 Department of Medicine, University of Mississippi Medical Center, 39213, Jackson, MS, USA; 3 Department of Medicine, Section on Endocrinology and Metabolism, Wake Forest University School of Medicine, 27157, Winston-Salem, NC, USA; 4 Jackson Heart Study, Jackson State University, 39217, Jackson, MS, USA; 5 Department of Epidemiology, University of Alabama at Birmingham, 35294, Birmingham, AL, USA; 6 NIDDK, National Institutes of Health, 20892, Bethesda, MD, USA; 7 NHLBI, National Institutes of Health, 20892, Bethesda, MD, USA

## Abstract

**Background:**

Adiponectin, paradoxically reduced in obesity and with lower levels in African Americans (AA), modulates several cardiometabolic risk factors. Because abdominal visceral adipose tissue (VAT), known to be reduced in AA, and subcutaneous adipose tissue (SAT) compartments may confer differential metabolic risk profiles, we investigated the associations of VAT and SAT with serum adiponectin, separately by gender, with the hypothesis that VAT is more strongly inversely associated with adiponectin than SAT.

**Methods:**

Participants from the Jackson Heart Study, an ongoing cohort of AA (n = 2,799; 64% women; mean age, 55 ± 11 years) underwent computer tomography assessment of SAT and VAT volumes, and had stored serum specimens analyzed for adiponectin levels. These levels were examined by gender in relation to increments of VAT and SAT.

**Results:**

Compared to women, men had significantly lower mean levels of adiponectin (3.9 ± 3.0 μg/mL vs. 6.0 ± 4.4 μg/mL; p < 0.01) and mean volume of SAT (1,721 ± 803 cm^3^ vs. 2,668 ± 968 cm^3^; p < 0.01) but significantly higher mean volume of VAT (884 ± 416 cm^3^ vs. 801 ± 363 cm^3^; p < 0.01). Among women, a one standard deviation increment in VAT was inversely associated with adiponectin (β = − 0.13; p < 0.0001) after controlling for age, systolic blood pressure, fasting plasma glucose, high-density lipoprotein cholesterol, triglycerides, education, pack-years of smoking and daily intake of alcohol. The statistically significant inverse association of VAT and adiponectin persisted after additionally adjusting for SAT, body mass index (BMI) and waist circumference (WC), suggesting that VAT provides significant information above and beyond BMI and WC. Among men, after the same multivariable adjustment, there was a direct association of SAT and adiponectin (β = 0.18; p = 0.002) that persisted when controlling for BMI and WC, supporting a beneficial effect of SAT. Insulin resistance mediated the association of SAT with adiponectin in women.

**Conclusion:**

In African Americans, abdominal visceral adipose tissue had an inverse association with serum adiponectin concentrations only among women. Abdominal subcutaneous adipose tissue appeared as a protective fat depot in men.

## Background

Obese individuals, particularly those with accumulated visceral fat, have reduced plasma levels of adiponectin [1], and are at increased risk for hypertension, type 2 diabetes and atherosclerotic events [2,3]. Adiponectin has been suggested to play an important role in atherosclerosis, endothelial inflammation, myocardial remodeling and several of the cardiometabolic risk factors [4,5]. Few studies have investigated the association of adiponectin with obesity (especially different fat deposits) in African Americans, known to have a lower plasma level of this adipokine [6-8].

Fat compartments have been shown to be differentially associated with the metabolic risk [9]. Variations in fat distribution mediate cardiometabolic risk factors [10]. Recent studies have shown that visceral adipose tissue (VAT) is more strongly associated with an adverse metabolic risk profile than subcutaneous adipose tissue (SAT) in both whites and African Americans [9-11]. We recently showed in the Jackson Heart Study (JHS) cohort of African Americans that VAT is a stronger correlate than SAT for most cardiometabolic risk factors, and that it remains significantly associated with these factors even after accounting for body mass index (BMI) [12]. Among obese Caucasians Framingham Heart Study (FHS) participants, SAT was a fat depot protective against high triglycerides levels [13].

The present study was designed to examine the associations of abdominal adiposity compartments with adiponectin in a large community-based sample of African Americans. The scientific hypothesis was that, similarly with white populations, VAT is more strongly inversely associated with serum adiponectin than is SAT. We aimed to determine if volumetric imaging methods of SAT and VAT provide information about adiponectin beyond that offered by simpler measures such as BMI and waist circumference (WC). We also queried if these associations are dependent on gender and if insulin resistance mediates the association of abdominal adipose tissues with adiponectin.

## Methods

### Study population

The JHS is a single-site, prospective cohort study of the risk factors and causes of heart disease in adult African Americans. A probability sample of 5,301 AA, aged 21 – 94 years, residing in a three county area surrounding the city of Jackson, MS, was recruited and examined at baseline (2000–2004) by certified technicians according to standardized protocols. Clinic visits and interviews occur approximately every three years. Annual follow-up interviews and cohort surveillance are ongoing. An overview of the JHS [14] and details of the study design and data collection methods [15] are published elsewhere. The present study included 2,799 participants who underwent multi-detector computed tomography (CT) measurements for visceral adiposity between 2007 and 2009 as part of the second JHS examination (JHS Exam 2) and had plasma adiponectin measured using frozen specimens stored at JHS Exam 1 (2000 – 2004). Written consent was obtained from each participant at the inception of the study, and the study protocol was approved by the Institutional Review Boards of the Morehouse School of Medicine and the participating JHS institutions: Jackson State University, Tougaloo College and the University of Mississippi Medical Center.

### Multi-detector CT scanning protocol for measuring adiposity

Overall, 4,203 participants attended the JHS Exam 2. Of these, 2,884 underwent multi-detector CT assessment for abdominal VAT and SAT. Participants were excluded from the CT scan exam if: 1) body weight was greater than 350 pounds (~160 kg) due to scanner limits; 2) pregnant or unknown pregnancy status; 3) female participants < 40 years of age; 4) male participant < 35 years of age. The research CT protocol included the heart and lower abdomen using a 16 channel multi-detector computed tomography system equipped with cardiac gating (GE Healthcare Lightspeed 16 Pro, Milwaukee, Wisconsin). Quality control and image analysis was performed at a core reading center (Wake Forest University School of Medicine, Winston-Salem, NC). The protocol included scout images, one ECG gated series of the entire heart, 110 and a series through the lower abdomen. The acquired abdominal imaging slices covering the lower abdomen from L3 to S1 were used for assessing VAT and SAT. Briefly, 24 contiguous 2-mm thick slices centered on the lumbar disk space at L4-5 were used for this analysis; 12 images before the center of the L4 - L5 disk space and 12 images after the disk space were used for quantification of VAT and SAT. The abdominal muscular wall was firstly manually traced and the fat volumes in different compartments were measured by semiautomatic segmentation technique. Secondly, Volume Analysis software (Advantage Windows, GE Healthcare, Waukesha, WI) was used to segment and characterize each individual voxel as a tissue attenuation of fat using a threshold range −190 to −30 Hounsfield units. The VAT and SAT volumes were the sum of VAT and SAT voxel over 24 slices. In our previous study, in a random selected sample of 60 participants, we found an interclass correlation coefficient for inter-reader comparisons of 0.95 for both VAT and SAT [12].

### Covariates

Covariate information was ascertained during the JHS Exam 1 (2000–2004). In all participants, the clinic visit included physical examination, anthropometry, survey of medical history and current medication use and collection of blood and urine specimens for biological assessment. In-clinic standing height and weight were measured in lightweight examination clothing without shoes or constricting garments. We calculated body mass index (BMI) as weight in kilograms divided by height in meters squared (kg/m^2^). Waist circumference (WC) was measured at the level of the umbilicus using a non-elastic tape measurer and rounded to the nearest centimeter. Education (as a measure of socioeconomic status) was categorized into four categories; less than high school (HS), HS graduate, more than HS but less than college and college graduate or more. Pack-years of smoking (defined as the number of years of smoking times the average number of cigarettes smoked per day divided by 20) were used as a variable in the multivariable models. Alcohol drinking (grams/day), also used in the multivariable modeling, was estimated from the frequency and portion sizes of beer, wine, and liquor ascertained from a validated 158-item food frequency questionnaire [16]. Menopausal status was queried through a self-reported questionnaire.

Lipid variables (high-density lipoprotein cholesterol, HDL-C and triglycerides, TG), fasting plasma glucose (FPG) and fasting insulin (FI) were measured using standard laboratory techniques. Insulin resistance status was estimated with the homeostasis model assessment, HOMA-IR, as (FPG x FI)/22.5 [17].

### Adiponectin measurement

Venous blood samples were withdrawn from each subject at baseline examination after more than 8 hours of fasting as described elsewhere [15]. Vials of serum were stored at the JHS central repository in Minneapolis, MN, at −80°C until assayed. Adiponectin concentration was measured in 2008 – 2012 as total adiponectin by an ELISA system (R&D Systems; Minneapolis, MN) [18]. The inter-assay coefficient of variation was 8.8%. No biological degradation has been described using stored specimens, indicating a high validity for our measurements [19].

### Statistical analysis

All analyses were stratified by gender to explore heterogeneities in the independent associations of adiponectin with the adiposity compartments. Both adiposity measures (VAT and SAT) were standardized by dividing them to their population standard deviation. Thus, all the regression coefficients were expressed per one standard deviation increase in VAT or SAT. Two-way interactions between adiposity measures and gender were formally tested by adding interaction terms in the fully multivariable adjusted models.

VAT and SAT were normally distributed; adiponectin values were positively skewed and normalized by logarithmic transformation (base 10). Age-adjusted Pearson correlations of VAT and SAT with adiponectin, the metabolic risk factors (systolic blood pressure, fasting plasma glucose, triglycerides, high-density lipoprotein (HDL) cholesterol, HOMA-IR), smoking and alcohol intake were performed. Multivariable regression models were constructed with VAT or SAT as the main independent variables (in separate and concomitant models) and adiponectin as the dependent variable. Models were generated in sequence by adding BMI, WC or both to the basic multivariable model that included age, systolic blood pressure, fasting plasma glucose, HDL-cholesterol, triglycerides, education, smoking and alcohol consumption. In order to contrast the added value of VAT and SAT above and beyond those of BMI and WC, we calculated and contrasted the models’ R-square, as explained variance. We also estimated the main variables associated with adiponectin (principal contributors to its variance) by using stepwise linear regression models in which HOMA-IR was added to the previous group of variables. The significance level cutpoint considered for entry into the stepwise forward-selection model was lower than 0.50. In order to assess the effect of weight change between the visits, we performed additional analyses with exclusion of participants that had more than one and a half BMI units change (which correspond to a 5 kg change in weight in a person 1.8 meters tall). We additionally conducted sensitivity analyses by excluding the participants on cardiovascular and lipid lowering medications, namely beta-blockers, angiotensin-converting-enzyme inhibitors, calcium-channels blockers and statins. All computations were performed by SAS software version 9.2 (SAS Institute Inc., Cary, North Carolina).

## Results

The study sample was comprised of 1,801 women (64%) and 998 men. The characteristics of the participants according to gender are presented in Table 1. The mean (standard deviation) age of the study sample was 55 (11) years for women and 54 (11) for men; it was 55 (11) years for the entire sample and thus similar with the entire JHS cohort [55 (13) years]. Men had significantly lower mean levels of adiponectin (3.9 μg/mL) than women (6.0 μg/mL; p < 0.0001). Men had significantly higher mean volumes of VAT as compared to women (884 cm^3^ vs. 801 cm^3^; p < 0.0001), whereas women had higher SAT volumes compared to men (2,668 cm^3^ vs. 1,721 cm^3^; p < 0.0001 for difference, Table 1). Statistically significant differences by gender were observed for the majority of the study covariates (including BMI). The distributions of fasting plasma glucose, systolic blood pressure and education did not differ by gender (Table 1).

**Table 1:** Characteristics (mean ± standard deviation) of study participants (N = 2,799)

	**Women (N = 1,801)**	**Men (N = 998)**	***P-value***
Age (years)	55 ± 11	54 ± 11	*.0004*
Adiponectin (μg/mL)	6.0 ± 4.4	3.9 ± 3.0	*< .0001*
VAT (cm^3^)	801 ± 363	884 ± 416	*< .0001*
SAT (cm^3^)	2,668 ± 968	1,721 ± 803	*< .0001*
BMI (kg/m^2^)	32.5 ± 6.8	29.4 ± 5.1	*< .0001*
WC (cm)	99.6 ± 16.1	100.3 ± 12.8	*< .0001*^*a*^
TG (mg/dL)	100.8 ± 65.2	109.9 ± 66.9	*.0007*
HDL-C (mg/dL)	55.3 ± 14.8	45.7 ± 11.7	*< .0001*
FPG (mg/dL)	97.3 ± 28.0	98.2 ± 26.7	*.47*
HOMA-IR	3.7 ± 2.3	3.3 ± 2.5	*.0002*
SBP (mm Hg)	125.7 ± 17.2	126.4 ± 16.6	*.25*
Education (≥ college)	38.7	37.3	*.11*
Smoking	4.3 ± 11.7	10.0 ± 19.3	*< .0001*
Alcohol intake	1.4 ± 5.7	8.8 ± 27.7	*< .0001*

^a^When compared as categorical variables defined with the US National Cholesterol Education Program Adult Treatment Panel III cutpoints criteria for abdominal obesity (88 cm in women and 102 cm in men);

VAT: visceral adipose tissue; SAT: subcutaneous adipose tissue; BMI: body mass index; WC: waist circumference; FPG: fasting plasma glucose in mg/dL; HOMA-IR: homeostasis assessment model - insulin resistance; SBP: systolic blood pressure in millimeters of mercury; TG: triglycerides in mg/dL; HDL-C: high-density lipoprotein-cholesterol in mg/dL; education: college education graduate or more (in percentage); smoking in packs-year and alcohol intake in grams/day.

### Correlations between obesity measures and adiponectin and other variables

Age-adjusted correlates of VAT and SAT with adiponectin and other covariates considered in our study are presented in Table 2. The correlation of adiponectin with abdominal adiposity measures was higher for VAT and higher for both VAT and SAT in women compared with men. In both genders, BMI and WC were correlated more strongly with SAT than VAT. HDL-cholesterol, triglycerides and fasting plasma glucose levels correlated more highly with VAT than with SAT, especially in women. The blood pressure values had low correlations with both VAT and SAT. BMI and WC had moderate correlations with the majority of cardiometabolic risk factors (Table 2).

**Table 2:** Age-adjusted Pearson correlation coefficients between adiponectin, VAT, SAT and covariates (N = 2,799)

	**Women (N = 1,801)**					**Men (N = 998)**				
	**Adiponectin**	**VAT**	**SAT**	**BMI**	**WC**	**Adiponectin**	**VAT**	**SAT**	**BMI**	**WC**
Adiponectin	---					---				
VAT	- 0.31^c^	---				- 0.17^c^	---			
SAT	- 0.16^c^	0.46^c^	---			- 0.10^b^	0.50^c^	---		
BMI	- 0.24^c^	0.50^c^	0.78^c^	---		- 0.21^c^	0.49^c^	0.74^c^	---	
WC	- 0.33^c^	0.57^c^	0.73^c^	0.81^c^	---	- 0.20^c^	0.58^c^	0.82^c^	0.84^c^	---
Smoking	- 0.003	0.04	- 0.04	- 0.04	- 0.01	0.02	0.06	- 0.07	- 0.09^a^	- 0.05
Alcohol intake	0.03	- 0.002	- 0.08^b^	- 0.08^b^	- 0.06^a^	0.09^a^	0.03	- 0.08^a^	- 0.09^b^	- 0.05
TG	- 0.27^c^	0.21^c^	- 0.003	0.09^c^	0.16^c^	- 0.27^c^	0.30^c^	0.13^c^	0.20^c^	0.23^c^
HDL-C	0.41^c^	- 0.21^c^	- 0.09^c^	- 0.19^c^	- 0.23^c^	0.36^c^	- 0.24^c^	- 0.23^c^	- 0.32^c^	- 0.33^c^
FPG	- 0.28^c^	0.23^c^	0.17^c^	0.22^c^	0.26^c^	- 0.14^c^	0.23^c^	0.15^c^	0.21^c^	0.26^c^
HOMA-IR	- 0.42^c^	0.38^c^	0.24^c^	0.35^c^	0.41^c^	- 0.28^c^	0.30^c^	0.28^c^	0.34^c^	0.40^c^
SBP	- 0.01	0.04	0.05	0.12^c^	0.08^b^	- 0.01	0.16^b^	0.05	0.13^b^	0.09^a^

^a^p < 0.05; ^b^p < 0.01; ^c^p < 0.001;

VAT: visceral adiposity tissue area; SAT: subcutaneous adipose tissue area; BMI: body mass index; WC: waist circumference; Adiponectin: logarithmically-transformed adiponectin plasma levels; p: p-values; HDL-C: high density lipoprotein cholesterol; FPG: fasting plasma glucose; HOMA-IR: homeostasis assessment model - insulin resistance; SBP: systolic blood pressure.

### Multivariable-adjusted regression models of adiponectin on VAT or SAT

Gender proved to be an effect modifier of both the association between serum adiponectin and VAT (p for interaction = 0.003) as well as for the association between adiponectin and SAT (p for interaction = 0.001). The results of the multiple linear regression analyses for the gender-specific association of VAT or SAT with log-transformed adiponectin are presented in Table 3. In women, a one standard deviation increment in either VAT or SAT was inversely associated with adiponectin (β = − 0.13 for VAT and β = − 0.07 for SAT) after controlling for age, systolic blood pressure, fasting plasma glucose, triglycerides, HDL-cholesterol, education, pack-years of smoking and daily intake of alcohol when these two adiposity measures were included separately in the models (models 1 and 2, respectively). The significant inverse association of VAT and adiponectin persisted after additionally adjusting for SAT, BMI or/and WC (model 3, 4, 5 and 6, respectively). The association of adiponectin with SAT became nonsignificant after accounting for BMI (model 7). Among our women participants, same results were obtained when adjusting additionally for menopausal status. Specifically, in the fully adjusted model that included BMI and WC (the equivalent of model 6), the inverse association between adiponectin and VAT was the same: β = − 0.08, p < 0.0001. Among men, there was a direct association of SAT and adiponectin (β = 0.18, 0.16 and 0.18; p = 0.002 to 0.0002) that was revealed when adjusting for BMI or/and WC (models 7, 8 and 9 in Table 3).

**Table 3:** **Multivariable-adjusted regression coefficients of VAT and SAT with adiponectin levels **^a^**by gender (N = 2,799)**

**Models**	**Covariates**	**VAT β Coefficient**	***P-value***	**Model’s R-square**	**SAT β Coefficient**	***P-value***	**Model’s R-square**
**Women**	**(N = 1,801)**						
Model 1	Basic^b^ + VAT	- 0.13	*< .0001*	0.24	---	*---*	---
Model 2	Basic + SAT	---	*---*	---	- 0.07	*< .0001*	0.22
Model 3	Basic + VAT, SAT	- 0.13	*< .0001*	0.23	- 0.02	*.32*	0.24
Model 4	Basic + VAT, BMI	- 0.11	*< .0001*	0.24	---	*---*	---
Model 5	Basic + VAT, WC	- 0.08	*< .0001*	0.25	---	*---*	---
Model 6	Basic + VAT, BMI, WC	- 0.08	*< .0001*	0.26	---	*---*	---
Model 7	Basic + SAT, BMI	---	*---*	---	- 0.001	*.97*	0.22
Model 8	Basic + SAT, WC	---	*---*	---	0.06	*.01*	0.25
Model 9	Basic + SAT, BMI, WC	---	*---*	---	0.05	*.05*	0.25
**Men**	**(N = 998)**						
Model 1	Basic^b^ + VAT	- 0.03	*.12*	0.16	---	*---*	---
Model 2	Basic + SAT	---	*---*	---	0.03	*.27*	0.17
Model 3	Basic + VAT, SAT	- 0.05	*.02*	0.17	0.06	*.05*	0.17
Model 4	Basic + VAT, BMI	- 0.02	*.29*	0.17	---	*---*	---
Model 5	Basic + VAT, WC	- 0.03	*.24*	0.17	---	*---*	---
Model 6	Basic + VAT, BMI, WC	- 0.03	*.24*	0.17	---	*---*	---
Model 7	Basic + SAT, BMI	---	*---*	---	0.18	*.002*	0.17
Model 8	Basic + SAT, WC	---	*---*	---	0.16	*.001*	0.18
Model 9	Basic + SAT, BMI, WC	---	*---*	---	0.18	*.0002*	0.18

^a^Log-transformed values;

^b^Basic multivariable model: covariates used for adjustment were age, systolic blood pressure, fasting plasma glucose, HDL-cholesterol, triglycerides, education (in four categories as described), smoking (pack-years) and alcohol intake (grams/day);

BMI: body mass index; WC: waist circumference; VAT: visceral adiposity tissue area; SAT: subcutaneous adipose tissue area.

Similar results with those presented in Tables 2 and 3 were obtained in the additional analyses with exclusion of participants with more than one and a half BMI units change between visits (N = 1,525). Specifically, among women, a one standard deviation increment in VAT was inversely associated with adiponectin (β = − 0.12, p < 0.0001) after multivariable adjustment that included BMI and WC (the equivalent model 6 from Table 3). Among men, a one standard deviation increment in SAT was directly associated with adiponectin (β = 0.08, p = 0.04) after multivariable adjustment that included also BMI and WC (the equivalent model 9 from Table 3).

Also, similar results were obtained when excluding participants on medications such as beta-blockers, angiotensin-converting-enzyme inhibitors, calcium-channels blockers and statins (N = 2,113). Specifically, among women, a one standard deviation increment in VAT was inversely associated with adiponectin (β = − 0.08, p = 0.0001) after multivariable adjustment that included BMI and WC (the equivalent model 6 from Table 3). Among men, a one standard deviation increment in SAT was directly associated with adiponectin (β = 0.17, p = 0.002) after the same multivariable adjustment (the equivalent model 9 from Table 3).

Table 4 presents the main variables associated with adiponectin levels according to gender, as indicated by the stepwise regression procedure using a glucose metabolism-related stage-modeling strategy. Insulin resistance showed minimal effect on the association of adiponectin with abdominal adiposity compartments. HOMA-IR mediated however the association of adiponectin with SAT among women. The main variance contributors for adiponectin were HDL-cholesterol and insulin resistance, in this order, in both genders (results not shown).

**Table 4:** **Stepwise regression stage-models of adiponectin**^**a **^**with glucose metabolism components by gender**^**b **^**(N = 2,799)**

	**Model without FPG**^**c **^**or IR**^**c**^		**Model with FPG**		**Model with IR**	
**Independent Variable**	**Estimate (β coefficient)**	***P-value***	**Estimate (β coefficient)**	***P-value***	**Estimate (β coefficient)**	***P-value***
**Women (N = 1,801)**						
**VAT**	**- 0.09**	***< .0001***	**- 0.08**	***< .0001***	**- 0.07**	***.0002***
**SAT**	**0.06**	***.02***	**0.06**	***.02***	**0.04**	***.09***
Age	0.01	*< .0001*	0.01	*< .0001*	0.01	*< .0001*
BMI	0.005	*.23*	0.005	*.22*	0.01	*.09*
WC	- 0.01	*< .0001*	- 0.01	*< .0001*	- 0.01	*< .0001*
SBP	0.001	*.29*	0.001	*.27*	0.001	*.12*
HDL-C	0.01	*< .0001*	0.01	*< .0001*	0.01	*< .0001*
TG	- 0.001	*< .0001*	- 0.001	*< .0001*	- 0.001	*.05*
Smoking	---	*---*	---	*---*	---	*---*
Alcohol Intake	---	*---*	---	*---*	---	*---*
FPG	N/A^d^	*N/A*^d^	- 0.0004	*.43*	N/A^d^	*N/A*^d^
HOMA-IR	N/A^d^	*N/A*^d^	N/A^d^	*N/A*^d^	- 0.06	*< .0001*
**Men (N = 998)**						
**VAT**	**- 0.03**	***.21***	**- 0.03**	***.21***	**- 0.03**	***.30***
**SAT**	**0.18**	***.0002***	**0.18**	***.0002***	**0.13**	***.004***
Age	0.01	*.0001*	0.01	*<.0001*	0.01	*.001*
BMI	- 0.01	*.008*	- 0.01	*.10*	- 0.02	*.01*
WC	- 0.01	*.12*	- 0.01	*.14*	---	*---*
SBP	0.001	*.26*	0.002	*.24*	0.001	*.37*
HDL-C	0.01	*< .0001*	0.01	*<.0001*	0.01	*< .0001*
TG	- 0.001	*.002*	- 0.001	*.002*	- 0.001	*.01*
Smoking	0.001	*.33*	0.001	*.28*	---	*---*
Alcohol Intake	0.001	*.11*	0.001	*.12*	0.001	*.11*
FPG	N/A^d^	*N/A*^d^	- 0.001	*.20*	N/A^d^	*N/A*^d^
HOMA-IR	N/A^d^	*N/A*^d^	N/A^d^	*N/A*^d^	- 0.05	*< .0001*

^a^Log-transformed values;

^*b*^*P-value* for sex by VAT interaction was 0.001 in the first model (without IR), 0.004 in the second model (with FPG), and 0.003 in the third model (with IR);

^c^By fasting plasma glucose (FPG) and/or HOMA-IR as continuous variables;

^d^N/A: not applicable (not used within the model);

The significance level considered for entry into the stepwise forward-selection regression model was 0.50.

BMI: body mass index; WC: waist circumference; VAT: visceral adiposity tissue; SAT: subcutaneous adipose tissue; SBP: systolic blood pressure; HOMA-IR: homeostasis assessment model - insulin resistance; HDL-C: high density lipoprotein-cholesterol; TG: triglycerides; smoking in packs-year and alcohol intake in grams/day.

Model’s R-square for the full model (without FPG or HOMA-IR): 0.29 in women and 0.18 in men; for the full model (with FPG): 0.26 in women and 0.18 in men; for the full model (with HOMA-IR): 0.32 in women and 0.21 in men.

### Differential association of VAT and SAT with adiponectin

**Figure 1:**
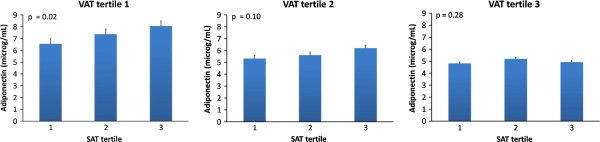
**Mean adiponectin levels in JHS women participants partitioned by tertiles of VAT and tertiles of SAT.** Subgroup sizes for each SAT tertile within VAT tertile were 200, 201 and 200 from the lowest to the highest. *Error bars* represent standard errors. *P-values* are given for linear trend in means of adiponectin levels.

**Figure 2:**
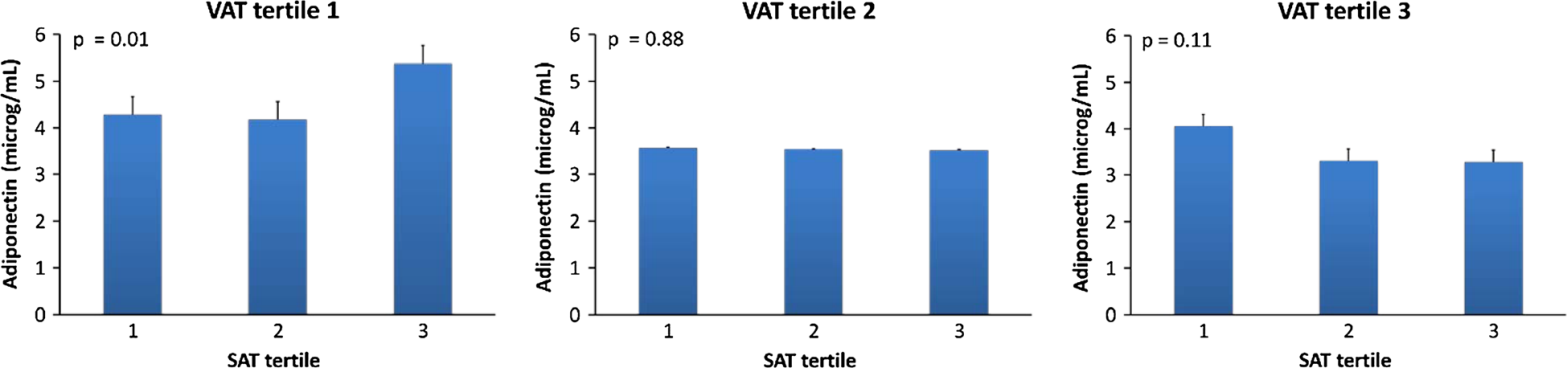
**Mean adiponectin levels in JHS men participants partitioned by tertiles of VAT and tertiles of SAT.** Subgroup sizes for each SAT tertile within VAT tertile were 111, 111 and 110 from the lowest to the highest. *Error bars* represent standard errors. *P-values* are given for linear trend in means of adiponectin levels.

In both genders, within the lowest VAT tertile, there was a statistically significant increase in adiponectin levels from the lowest to the highest SAT tertile (Figures 1 and 2). No such increase was documented within the highest two VAT tertiles.

## Discussion

### Principal findings

Our study carried out in a large population-based sample of African Americans showed that volumetric CT-scan measures of VAT and SAT were associated with adiponectin depending on gender. In women, the significant inverse association of VAT and adiponectin persisted after additionally adjusting for body mass index (BMI) and waist circumference (WC), suggesting that VAT provides significant information above and beyond BMI. Moreover, the significant inverse association with adiponectin persisted after including SAT into the model. In men, SAT had a significant direct association with adiponectin that persisted after including BMI and WC to the model. In both genders, in the lowest VAT tertile, there was an increase in adiponectin levels from the lowest to the highest SAT tertile. It is notable that, in contrast to African American women, VAT was not a significant predictor of adiponectin levels in African American men. Our study confirms insulin resistance as a mediator for the association of adiponectin with subcutaneous abdominal adiposity compartment in women.

### In the context of previous literature

Our results are consistent with the observation that men are prone to abdominal fat deposition, particularly in the abdominal cavity, a condition described as visceral obesity [20]. Notably, despite known differences in body fat distribution between individuals of African and European ancestry [21,22], we found similar gender interactions with VAT and adiponectin levels as in previous studies [8]. Similarly, gender proved to be an effect modifier of the association between SAT and serum adiponectin [23]. Nevertheless, opposite to previous studies that indicated an inverse association between SAT and adiponectin [23], our investigation suggested a direct association between these two variables, confirming a series of previous investigations [24,25]. Among men, the nonsignificant association between VAT and adiponectin provides insight which might explain why when compared with other races/ethnicities African American men have lower adiponectin levels despite smaller VAT compartments.

Waist circumference cannot differentiate between VAT and SAT compartments despite the fact that WC appears as a good predictor of VAT [26]. This is particularly important, as VAT and SAT may be differently associated with inflammatory cytokines secretion and with differential metabolic risk profiles [27]. The negative correlation between adiponectin and VAT is stronger than with SAT [28,29]. In 69 non-diabetic African Americans, adiponectin negatively correlated with VAT (r = −0.41) in men and with VAT (r = −0.55) and SAT (r = −0.35) in women [8]. In findings similar to our own, Considine and colleagues found that SAT was not significantly associated with adiponectin in the models that adjusted concomitantly for VAT [8].

Recent Jackson Heart Study (JHS) data suggest that abdominal obesity is particularly profound among African Americans as indicated by the fact that nearly two-thirds of the JHS sample has an elevated WC [30]. Whether compared with whites, African Americans with elevated WC experience a higher risk for cardiovascular events due to greater abdominal adiposity is uncertain because whites have greater VAT than African Americans [31] and, in nationwide samples, higher WC [32]. At similar degrees of obesity as defined by BMI, African Americans have a lower quantity of VAT compared with whites and Asian populations, despite higher rates of insulin resistance, diabetes and hypertension [33].

VAT secretes less adiponectin than SAT [34]. Low levels of adiponectin, high levels of leptin and chronic low-grade inflammatory state are generally observed in the obese status and have been associated with insulin resistance and metabolic syndrome [2]. Preliminary clinical evidence has linked these adipose tissue-derived hormones to measures of atherosclerosis and to development of future cardiovascular events [35], suggesting that adiponectin, leptin and C-reactive protein (CRP) may play an important role for increased risk of cardiovascular mortality in individuals with the metabolic syndrome. In the few studies conducted so far, higher WC was associated with lower total adiponectin after controlling for the effect of total fat mass, indicating that these associations are driven mainly by central fat accumulation, as indicated by the WC level rather than by total body adiposity [36,37]. As shown by Steffes and colleagues in the CARDIA cohort (3,355 study participants), central obesity, as measured by WC, is a primary factor affecting levels of circulating adiponectin [38]. Similarly with our investigation, both SAT and VAT were strongly correlated with insulin resistance (but with gender differences) in an investigation conducted among 78 nondiabetic African Americans [39]. Moreover, as shown by Tulloch-Reid and colleagues [39], among African American women SAT have a greater effect on insulin resistance, in agreement with our findings.

Few previous studies have assessed the association of adiponectin with abdominal adipose tissue compartments as measured by CT scan. In a study conducted in nondiabetic women, adiponectin was more strongly determined by VAT, whereas leptin was more strongly influenced by SAT [23]. In an investigation conducted in a Japanese male population, adiponectin levels were inversely associated with VAT and directly associated with SAT when these two measures of abdominal adiposity were both in a regression model [25]. In this study from Japan, the negative regression slope of the association between VAT and adiponectin (the steepness of the association) was four times larger compared with that in our African American population. Considering also the lower VAT volumes (about 75% lower than those in the white participants in the Framingham Heart Study) and lower adiponectin values in African Americans compared with white and Asian populations, this suggests that the VAT – adiponectin relationship is less strong in African Americans men compared with other populations. In the same direction, in a sample of participants selected from the IRAS Family Study, sample that included 522 African-Americans, adiponectin was significantly associated with age, gender, HDL-cholesterol, VAT and SAT [24]. The slope of the association between adiponectin and VAT was more than twice compared with the value that we detected within our investigation. It is worthy to note that the mean values of adiponectin within our sample were lower compared with the IRAS Family sample as well as with those from the Health ABC study that reported an association between adiponectin and risk of coronary heart disease [40]. Our results showing in women a higher slope of the association between VAT and adiponectin (when contrasted with that between SAT and adiponectin) are in agreement with a series human adiposity tissues studies that indicated a lower adiponectin secretion in omental fat versus subcutaneous fat [41].

### Potential mechanisms

Although traditionally regarded as a silent organ that passively stores excess energy, the adipose tissue is now considered an endocrine organ not only contributing to the management of energy flux within the body but also interacting with the inflammatory system and the vascular wall. Several studies have demonstrated that adipose tissue actively produces a variety of locally and systemically functioning bioactive molecules that interact in various obesity-related diseases, e.g., leptin [42], adiponectin [43], tumor necrosis factor-α (TNF-α) [44], plasminogen-activator inhibitor type-1 (PAI-1) [45] and resistin [46]. Adiponectin was discovered to be the most abundant adipose-specific transcript [43]. Furthermore, recent studies have underlined that there are intricate interplays among adipocytes, the sympathetic nervous system and the renin-angiotensin system, which participate in the obesity-associated dysmetabolic state [47]. The possible explanation for differences between VAT and SAT may be related to differences in their anatomic location and cytokine secretion profiles. SAT may preferentially release more leptin, whereas VAT may mainly release tumor-necrosis factor-α, known to influence the secretion of adiponectin [4]. In addition, a series of studies showed that androgens reduce plasma adiponectin [48], providing insights into the gender differences of the association between VAT and adiponectin. Additionally, adiponectin’s compensatory effects of metabolic disorders’ improvements seem to entail massive expansion of subcutaneous adipose tissue depot [49].

### Implications

Despite a lower amount of VAT and a lower concentration of serum adiponectin in African Americans, the inverse association in women of visceral adiposity with adiponectin that we showed extends the findings from studies among participants of European ancestry [9,11]. The different magnitude of the association supports the need for further studies to assess the mechanisms of this association and to prospectively link it to cardiometabolic risk. Among men, the significant direct association of adiponectin with SAT supports the postulated beneficial effect of SAT (i.e., that SAT might be a buffer mechanism that acts initially to compensate for an increase in WC) [13,50].

### Strengths and limitations

The main strength of our investigation is that this study is among the first on the topic with a very large community-based sample of African Americans. Another strength of this study is the precise measurement of abdominal VAT and SAT volumes using advanced imaging techniques. Among the limitations of our investigation is the fact that adiponectin and visceral adiposity were measured at different points in time. This is partially mitigated by the fact that similar results were obtained when excluding participants with significant body mass change between the study visits (as our sensitivity-type of analyses indicate). Another inherent limitation is that generalizability to other ethnic group cannot be applied.

## Conclusions

In African Americans, VAT had an inverse association with serum adiponectin concentrations only among women. Among men, SAT had a direct association with adiponectin supporting a buffer mechanisms to compensate for an increase in waist circumference. Considering the protective role of adiponectin for several cardiometabolic endpoints, the reduction of visceral fat in African American women might be an essential preventive measure for metabolic syndrome and its consequence, cardiovascular disease.

## Competing interests

The authors declare that they have no competing interests.

## Authors’ contributions

AB, JL, AES and GHG conceived of and designed the study. AB and DAH performed the statistical analyses. AB, JL, DAH, AES and GHG interpreted the results. AB and GHG drafted the manuscript. All authors revised the manuscript for intellectual content, and read and approved the final manuscript.

## Pre-publication history

The pre-publication history for this paper can be accessed here:

http://www.biomedcentral.com/1471-2261/13/9/prepub
